# Virtual Water Trade in the Service Sector: China’s Inbound Tourism as a Case Study

**DOI:** 10.3390/ijerph18041769

**Published:** 2021-02-11

**Authors:** Yu Zhang, Jin-he Zhang, Qing Tian

**Affiliations:** 1 School of Geography and Tourism, Qufu Normal University, Rizhao 276826, China; zhangyu_0730@163.com (Y.Z.); qingtian@qfnu.edu.cn (Q.T.); 2 Huangshan Park Ecosystem Observation and Research Station, Ministry of Education, Huangshan 245800, China; 3 School of Geography and Ocean Science, Nanjing University, Nanjing 210023, China

**Keywords:** virtual water export, tourism water footprint, spatial-temporal evolution, structural difference, water resource management, China inbound tourism

## Abstract

Research on virtual water and the water footprint is mainly focused on agriculture and industry, and less so on the service sector. The trade in products generates virtual water flow, as does the flow of people. The flow of international tourists will inevitably lead to the transfer and exchange of water resources embedded in the virtual form. This study takes China’s inbound tourism flow as the research object, from the perspective of the water footprint, in order to explore virtual water “exports” to the world. Based on kernel density estimation and ArcGIS spatial analysis, spatial-temporal evolution and structural difference were investigated. Virtual water “exports” showed an increasing trend. The kernel density estimation curves basically exhibited a “single peak” feature which indicated that virtual water “exports” from tourism were not significantly polarized in China. In terms of spatial evolution, this varied greatly at the provincial and regional level and Guangdong was always in the high value area. The south displayed greater values than the north, but this difference in provinces narrowed over the years. The water footprint of food was the largest, more specifically, the green component of this water footprint. Promoting a reasonable diet, reducing food waste, improving agricultural production technology, reducing the frequency of changing hotel supplies, and encouraging the use of new energy helped to reduce the water footprint. Virtual water trade in the service sector provides a new idea for helping to mitigate the global water crisis, in addition to virtual water flow for agricultural products.

## 1. Introduction

Water is the basis of the survival of all living beings. It is a key factor in maintaining human daily life, maintaining a balanced ecosystem and promoting social and economic development. Due to the development of the world economy, climate change, and population growth, the world is facing a serious problem regarding water scarcity. This water resources problem has seriously restricted economic and social development in many countries or regions in the world, because of the prominent contradiction between water supply and demand, coupled with low level and ineffective water resources management and poor water governance, etc. In the Middle East and the Sahel, for example, water stress is causing extreme social stress and the World Bank predicts that the economic impact of water scarcity will be significant, affecting 6% of its GDP by 2050 [1]. The fact that the world is on the brink of a global crisis has raised awareness of the problem and prompted countries around the world to take action. We must be deeply aware that the supply of water resources is a long-term social task that depends on the sustainable, comprehensive, flexible and targeted application of various political and technological means.

At present, the study of problems in regional water resources is made from the perspective of physical water resources which has certain limitations. Virtual water and water footprint are new concepts, which broaden our understanding of water resources and are considered effective tools for measuring the amount of water used to produce each of the goods and services we use [2]. In 1993, when Tony Allan was explaining the water problem of the Middle East, he innovatively proposed the concept of “virtual water” to describe the amount of water consumed in the production of a product or service. Virtual water is different from physical water resources. The characteristics of virtual water are “virtual” and “invisible”, also known as embodied water, embedded water and invisible water [3,4]. The concept of the water footprint, which is based on the concept of virtual water, was proposed by Hoekstra in 2002. This refers to the amount of water resources needed to produce the products and services consumed by a certain group of people under a certain material living standard and represents the real amount, including physical water and virtual water [5,6]. According to source, water can also be divided into green, blue and gray water footprint [7,8], as well as the costed-based quantitative water footprint (WFqt) and the qualitative water footprint (WFql) [9]. The water footprint can truly describe the demand and impact of human production and consumption activities on water resources of different spatial and temporal attributes. Virtual water and the water footprint have become the focus in water resource studies, and research is mainly concentrated on global [7,8,10,11,12], regional [13,14,15], national [16,17,18,19] and city level [20] virtual water trade and water footprint evaluation that is predominantly focused on agriculture and industry, such as cotton [12], milk [21], rice [22,23], maize [24], tea and coffee [25], potato [26,27], sugarcane and cassava [28], fuel production [29], bioenergy production [30,31,32,33], electricity generation [34,35,36,37], wine production [38,39], cosmetics [40], textile production [41], and human and veterinary pharmaceuticals [42]. However, studies on the service sector need to be enriched, especially the tourism industry which is an important part of the service sector and the most active factor.

Both regionally and globally, tourism is a significant water consumer and a high-value industry for water resources. Studies of water consumption and the management of tourism have usually conducted evaluations in the context of environmental indicators [43]. In addition, research focused on water use of tourism infrastructure, such as hotels, swimming pools, golf, spas, water parks and other water resources, has focused more on direct use [44]. Gössling stressed the importance of both the direct and the indirect water use of tourism. Comprehensive water resource indicators should include local water availability, direct and indirect water use, planning and management [44,45]. The concept of the tourism water footprint comprehensively considers the direct and indirect water consumption of tourism, which is helpful for revealing and evaluating the real possession and consumption of water resources for tourism development and promoting sustainable development. It has been defined as the amount of water needed to satisfy the consumption of products and services by international and domestic tourists [46]. However, research on the tourism water footprint is still in its infancy, and there are few studies in the literature. In 2012, Professor Stefan Gössling systematically discussed the direct and indirect water consumption involved in tourism activities, and incorporated the tourism food, infrastructure and energy consumption into the total tourism water consumption [45]. Later, scholars began to study the water footprint of tourism on different regional scales and in different tourism departments, such as five tourist destinations in the eastern Mediterranean [47]; Spain’s tourism industry [48]; the Chinese tourism industry [49]; a resort hotel on Lodz Island in Greece [44]; the Liming Scenic Area of Yunnan, China [50]; Huangshan Scenic Area [51,52,53]; Honghe Hani Terraces [54]; the back-mountain of Qingcheng [55]; Sanya city [56]; and Wuhan city [57].

Additionally, mobility is one of the main features of modern society. Mobility has become an effective channel for exploring the rational allocation of resources at the global level. Tourism is a large-scale human flow phenomenon; it can cause the interregional agglomeration and diffusion of material flow, information flow, culture flow, and energy flow and can have a positive or negative impact on the social economy, resources, and environment of specific regions. International tourism is a global-scale flow, and such cross-border flows on a global basis will inevitably bring about the transfer and exchange of water resources embedded in the virtual form, and have an impact on the spatial distribution of global water resources. In addition to the trade in and the flow of virtual water for agricultural products, the mobility of people, especially the virtual water flow involved in tourism, gives us an idea of the volumes of water incorporated in that productive activity. Then we can take actions and make decisions to alleviate the global water crisis.

Inbound tourism plays an important role in the development of China’s tourism industry, not only generating economic development, but also affecting the ecological environmental system, including water resources. In terms of trade, virtual water trade takes place as the volume of water embedded in the products or services exchanged internationally [58]. With the import and export of products or services, virtual water is imported and exported. The development of China’s inbound tourism is essentially a service export. The consumption of water resources by inbound tourists in China is equivalent to China’s “exports” of water resources, representing China’s contribution to global water resources.

Based on the water consumption of tourism and the mobility of tourists, the objective of this study was to measure and analyze the virtual water “exports” generated by China’s inbound tourism from 2001 to 2018, including spatial-temporal evolution and structural difference. Kernel density estimation and ArcGIS were used to analyze the temporal variation and spatial differences between provinces and regions, and deeply analyze spatial evolution. Structural differences were mainly discussed in terms of accounts and water resource types. Water footprint theory is applied to inbound tourism in the service sector, offering a new perspective for understanding. This is the first time that the water footprint of inbound tourists has been measured on a national scale, which provides a reference for future policies on inbound tourism development. The flow of virtual water for tourism provides a new idea to help alleviate the global water crisis.

## 2. Methods and Data

### 2.1. Methods

The water footprint of inbound tourists (ITWF) in China is the sum of the water footprints of inbound tourists received in all provinces. The research scale was relatively large, and there were great difficulties in obtaining precise data, such as data on visiting, shopping, entertainment, water for management and sewage, etc. Either there were no statistics, or they were difficult to obtain. Therefore, based on contribution to the overall water footprint and data acquisition, this study only estimated water footprint accounts that displayed a significant contribution to the overall tourism water footprint. In the measurement of the water footprint of Mount Huangshan, it was found that the food water footprint accounted for the highest proportion, up to 59.86%, followed by the transportation and accommodation water footprint [52]. In the measurement of the tourism water footprint of Wuhan, it was found that the food water footprint accounted for the highest proportion, up to 37%, followed by the water footprint of visiting, transportation, and accommodation [57]. Hadjikakou believes that the water footprint of tourists’ food consumption contributed the most to the overall tourism water footprint [47]. It can be seen that the water footprint of tourism food consumption, transportation and accommodation represents the sectors that contribute the most to the overall tourism water footprint; these sectors are usually included in tourism water footprint accounting. Therefore, the water footprint of inbound tourism (ITWF) presented in this article covers the water footprint of food consumption ($ITWF_{food}$), accommodation ($ITWF_{accom}$) and transportation ($ITWF_{trans}$) in relation to inbound tourists.

$$ITWF=ITWF_{food}+ITWF_{accom}+ITWF_{trans}$$

(1) The food water footprint of inbound tourists.

The flow of tourists generates a virtual water flow. Inbound tourists consume virtual water embedded in Chinese food, which is equivalent to a virtual water export and has an impact on global water resources. The food water footprint of inbound tourists is specific to each province, and is defined as the food virtual water consumed by each province when receiving inbound overnight tourists, including foreigners and tourists from Hong Kong, Macao and Taiwan, China. This requires data on inbound tourists’ food consumption and the water footprint per unit product. However, there are no statistics available for tourist food consumption. Therefore, in this study, the food consumption data of inbound tourists was related to the food consumption of permanent residents in each province, and the per capita value was obtained based on the consumption of urban and rural residents. Then, data could be calculated based on the average stay time and number of inbound tourists.

(2) $$ITWF_{food}=\sum_{i=1}^{n}\left(W_{i}*Q_{i}\right)\;$$

$W_{i}$ is the water footprint of per unit food i (m^3^/t);$\;Q_{i}$ is the daily consumption of food i (t)the tourists consumed.

(2) The accommodation water footprint of inbound tourists.

The measurement of the accommodation water footprint requires the average daily water consumption data of inbound tourists and accommodation days, including direct and indirect water consumption. Tourism data do not include statistics of this kind. Therefore, individual behavioral differences were ignored in this study, and a unified standard was adopted for the average daily accommodation water footprint of inbound tourists; that is, direct water consumption of 0.35 m^3^ per guest per night, and virtual water consumption implied by energy utilization related to hotel accommodation of 0.075 m^3^ per guest per night [59]. The total average daily accommodation water footprint was 0.425 m^3^ per guest per night. In China, inbound tourists usually choose to stay in high-star hotels, and hotels of the same star rating are basically the same in terms of facilities and services. Moreover, according to the “Code for Design of Building Water Supply and Drainage” GB 50015-2003 (2009 edition), the maximum daily living water quota for tourism in hotel rooms is 0.25–0.40 m^3^ per bed per day. Therefore, in this study, it was appropriate to adopt the standard of direct plus indirect water consumption of 0.425 m^3^ per guest per night for the daily accommodation of inbound tourists. Then, according to the average stay time and number of inbound tourists, the accommodation water footprint of inbound tourists could be calculated.

(3) The transportation water footprint of inbound tourists.

The transportation water footprint mainly consists of the virtual water consumption generated by energy consumption. Energy demands and embodied water use for transport are related to transport distances and the transport modes used. The purpose of this study was to measure the water footprint of China’s inbound tourism, which required the summation of the data for each province. There are no relevant statistics on the transportation mode, travel distance and flow direction of inbound tourists for each province, so we could not accurately measure this aspect. Therefore, in this study, we adopted a unified standard for the transportation water footprint of inbound tourists, namely, 0.13 m^3^ per guest per night [59]. The data were obtained from the travel and distance exhibited by tourists for each mode of transportation around the world, which was based on the Dynamic Global Tourism Traffic Model (GTTM^dyn^) and taking travel costs, travel time, income distribution, per capita GDP, and population for both domestic and international tourism into account [60]. Then, the transportation water footprint could be calculated according to the average stay time and number of inbound tourists.

In addition to the model of water footprint of inbound tourists (ITWF), kernel density estimation and ArcGIS spatial analysis have been used. Kernel density estimation is one of the most effective tools for studying the state of the spatial disequilibrium distribution. It is mainly used to estimate the probability density of random variables and describe the distribution state of random variables with a continuous density curve. In this study, it was used to analyze the temporal dynamic evolution of the water footprint of inbound tourists. The spatial analysis tools that are part of the ArcGIS system permit the spatial location, distribution, form, formation and evolution of geographical objects to be obtained and analyzed from spatial data. This helps us to determine whether the patterns that we see are significant, conduct an imagery analysis to detect change over time, and make important decisions using more than a simple visual analysis.

### 2.2. Data

The water footprint of inbound tourists was measured on three accounts, including food consumption, accommodation, and transportation (Table 1). The water footprint of food consumption required data on the food consumption of inbound tourists, the water footprint of each agricultural products unit, the number of inbound tourists and the number of days tourists stayed for. There are no relevant statistics on tourists’ food consumption in China. For tourists, who generally have the habit of ‘doing as the Romans do’, tasting the destination’s characteristic food is also one of the essential aspects of tourism [61]. Therefore, the consumption of the permanent residents of each province was adopted as the reference. The data mainly involved the number of permanent residents in cities and towns of the various provinces and the food consumption data of urban and rural residents from 2001 to 2018; the average value calculated on this basis was used as the food consumption of inbound tourists. The data were obtained from the China Statistical Yearbook, Statistical Yearbook of Provinces [62], and Statistical Bulletin of National Economy and Social Development of Provinces. It should be noted that the data included 31 provincial administrative regions in China, excluding Hong Kong, Macao and Taiwan.

The water footprint of agricultural products includes the water footprints of crop products and animal products. The standard data were obtained from reports no. 47 and 48 on water footprints of unit agricultural products published by Mekonnen and Hoekstra [7,8]. Report 47 provided the water footprint of unit crop products, covering the water footprint of different products of various countries and subordinate administrative regions, and report 48 provided the water footprint of animal products per unit, covering the average value of the water footprint of livestock products of all countries globally. Data on the number of inbound tourists and the number of days stayed for each province were obtained from the China Tourism Statistical Yearbook [62]. The water footprint of accommodation and transportation and the daily consumption per capita were average data.

## 3. Results

### 3.1. Temporal Variation of Virtual Water “Exports”

#### 3.1.1. General Analysis

**Figure 1:**
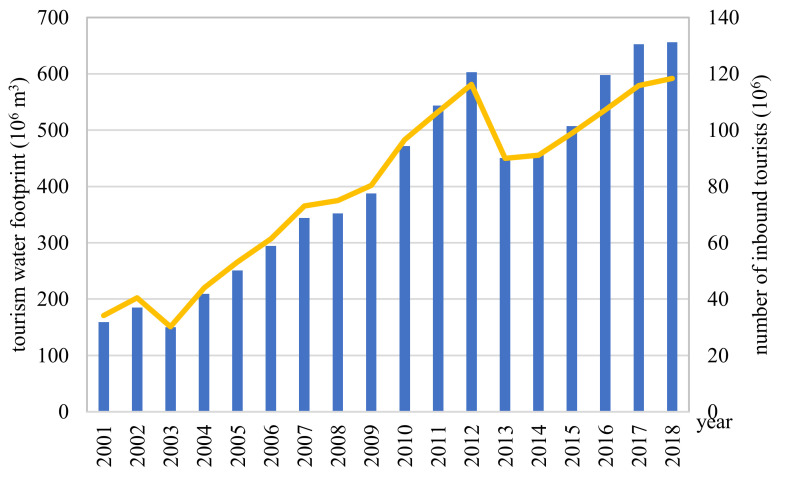
Water footprints (left axis) and number of inbound tourists (right axis) to China from 2001 to 2018.

Inbound tourists consume Chinese food, stay in Chinese hotels, and use various means of transportation in China. In addition to direct water consumption, virtual water hidden in food, accommodation, and transportation is also consumed, which is equivalent to China’s ‘exports’ of water resources. After calculation, the total water footprint, namely the virtual water “exports” for Chinese inbound tourists from 2001 to 2018 was 7273.15 × 10^6^ m^3^, with an annual average of 404.06 × 10^6^ m^3^. Overall, this showed an upward trend, from 159.02 × 10^6^ m^3^ in 2001 to 656.27 × 10^6^ m^3^ in 2018, with an increase rate of 312.70%. This was basically in line with the overall trend of the number of inbound tourists in China, which more than tripled between 2001 and 2018. The number of inbound tourists and the trend in the tourism water footprint are shown in Figure 1. As can be seen from the figure, both exhibit a rising trend, and the change trend is basically the same. Virtual water “exports” from inbound tourists were closely related to the number of inbound tourists. According to the multi-year average tourism statistics from 2001 to 2018, the main source countries can be divided into seven regions, namely, Hong Kong, Macao, and Taiwan; Japan and South Korea; Southeast Asia and South Asia; Russia and others; North American countries; European countries; and Oceanian countries [63]. Among the foreign entry markets, Japan and South Korea were the main source markets. The data also showed that the virtual water “exports” generated by the development of inbound tourism mainly flowed to Japan and South Korea. This only took the water footprint of food consumption, accommodation and transportation into consideration, and the result was relatively small. However, for more than 200 countries in the world, the annual export of 404.06 × 10^6^ m^3^ of water resources is already a figure that cannot be reached by most countries that trade with China in agricultural products. For example, the virtual water embedded in China’s agricultural exports to Nepal, Chile, Norway, Kenya, Tajikistan, Denmark, New Zealand, Hungary, Sweden, the Czech Republic, Finland, and Austria was less than 404.06 × 10^6^ m^3^ per year [16].

The water footprint of inbound tourists fell twice between 2001 and 2018, in 2003 and 2013, respectively (Figure 1). Accordingly, the period can be roughly divided into three development stages, roughly: 2001–2002 was the first stage; the second stage was from 2003 to 2012; and the third stage was from 2013 to 2018. In 2001, the global tourism industry experienced negative growth due to the impact of terrorist attacks and a military attack. Inbound tourism to China was also affected, growing only slowly. The second stage was from 2003 to 2012. In 2003, severe acute respiratory syndrome (SARS), a new high-risk infectious disease caused by an unknown coronavirus, spread in China and around the world. This was a disaster for the health of the Chinese public, and thus resulted in a huge loss to China’s tourism industry. The outbreak of SARS caused foreign exchange losses of inbound tourism amounting to US $5.67 billion [64]. After 2003, China’s inbound tourism grew steadily, and the water footprint of inbound tourists showed an increasing trend. The third stage was from 2013 to 2018. The decline in 2013 was mainly influenced by destination factors and exogenous factors, among which environmental change and the exchange rate were most important. In addition, we must realize that China’s inbound tourism market has declined significantly since 2020, having been affected by the global Corona Virus Disease 2019 (COVID-19) epidemic. For the sake of safety, potential inbound tourists will shorten their travel distance and the surrounding local market will become the main market in the early stage of inbound tourism recovery. Therefore, during the epidemic period and recovery period, China’s inbound tourism water footprint will be significantly reduced, not only in terms of inbound tourism, but also of domestic tourism.

#### 3.1.2. Inter-Annual Dynamic Evolution

**Figure 2:**
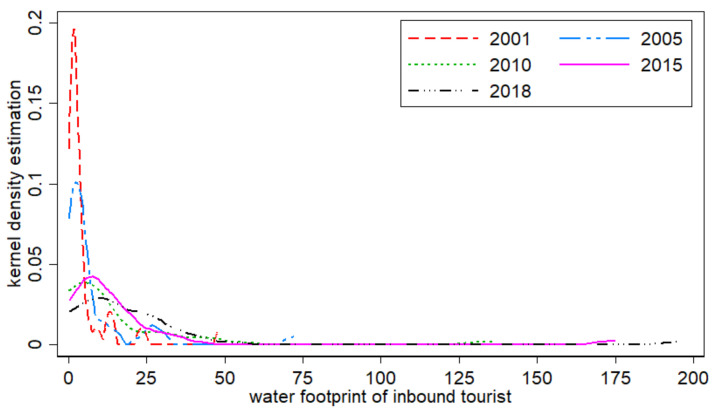
The dynamic evolution of the water footprint of inbound tourists to China from 2001 to 2018.

Due to the differential development of economic, transportation, and resource endowments, the development of inbound tourism in each province will change over time. Kernel density estimation is employed to trace the distribution state of random variables by using a continuous density curve, which can better capture the distribution characteristics of random variables. The period 2001–2018 involves China’s four five-year plans, from the Tenth Five-Year Plan to the 13th Five-Year Plan. The years 2005, 2010 and 2015 are the end years of the five-year plans respectively, reflecting the achievements of the whole five-year plan. In addition, 2001 and 2018, represent the starting and ending years of the study. Therefore, we chose the virtual water “exports” in the years 2001, 2005, 2010, 2015, and 2018 to analyze the inter-annual dynamic evolution (Figure 2).

It can be seen from the figure that, in terms of shape, the water footprints of inbound tourists from 2001 to 2018 basically show a “single peak”-type feature, indicating that the water footprints of inbound tourists in China were not significantly polarized. However, there was a big difference in some years. In 2001 and 2005, the kernel density estimation curve’s main peak and side peak coexisted, indicating that there was a big difference among provinces, and serious polarization. After 2010, the kernel density curves all showed a single peak shape, indicating that the differences between provinces were decreasing. In terms of location, the kernel density estimation curves presented an obvious skewed distribution, with a shorter peak on the left and a longer trailing tail on the right. Moreover, after 2010, the peak gradually moved to the right, indicating that, with the evolution of time, the water footprint of inbound tourists presented an increasing trend. Provinces with small water footprints have achieved relatively faster development, narrowing the development gap with other provinces. In terms of the peak value, the height of the peak generally declined, and the shape evolved from a steep state to a flat state, while the width of the peak increased with time. The peak value experienced a sharp decline to a flat and stable state, a sharp decline from 2001 to 2010, and then a flat and stable state. Generally speaking, after 2010, the differences between provinces in terms of the water footprint of inbound tourists gradually narrowed.

### 3.2. Spatial Variation of Virtual Water “Exports”

This section analyzes the differences in the virtual water “exports” generated by inbound tourists in the provinces, including the eight regions of China, located in both the south and north, with three economic zones, and analyzes the spatial evolution of the water footprints of inbound tourists.

#### 3.2.1. Provincial Differences

The virtual water “export” generated by inbound tourists varies greatly at the provincial level. From 2001 to 2018, the overall water footprint of inbound tourists in Guangdong province was the highest, reaching 2153.66 × 10^6^ m^3^, with an annual average of 119.65 × 10^6^ m^3^. According to tourism statistics, Japan, the United States, and South Korea were the main sources of foreign tourists in Guangdong [65]. The resulting virtual water exports also mainly flowed to these three countries, representing about a quarter of the exports. Beijing and Jiangsu were next, with an annual average of 32.47 × 10^6^ and 32.29 × 10^6^ m^3^, respectively. Virtual water also mainly flowed to Japan, the United States and South Korea. In addition, the water footprint of inbound tourists from the three provinces and cities accounted for 46% of the country’s total. The areas with the lowest values were Ningxia, Qinghai, and Gansu, where the average annual water footprint of inbound tourists was 0.13 × 10^6^, 0.27 × 10^6^ and 0.37 × 10^6^ m^3^, respectively. Guangdong province, which had the largest water footprint, and Ningxia, which had the smallest, exhibited a gap of 920.38 times when compared. The water footprint of inbound tourists in most provinces showed an upward trend, with a growth multiple of between 0.44 and 15.28. However, the water footprint of inbound tourists in Gansu province showed a downward trend, from 0.54 × 10^6^ m^3^ in 2001 to 0.26 × 10^6^ m^3^ in 2018. In addition, the water footprint of food consumption, accommodation and transportation of inbound tourists in Gansu decreased simultaneously. According to the calculation process, the reason for this may be the decrease in the number of inbound tourists, from 0.22 million in 2001 to 0.1 million in 2018.

#### 3.2.2. Regional Differences

Geographically, China’s 31 mainland provinces, cities and autonomous regions can be divided into eight regions, including northeast China, North China, the Huang-Huai-Hai Region, northwest China, southeast China, the middle and lower Yangtze River region, the South China region, and the southwest China region. After calculation, the contribution rate of the water footprint of inbound tourists in South China was the largest among the eight regions. From 2001 to 2018, the total amount was 2360.63 × 10^6^ m^3^, with an average annual rate of 131.15 × 10^6^ m^3^, accounting for 32.46% of the total water footprint of inbound tourists in China. South China also received the highest number of inbound overnight tourists in China, receiving 544.84 million person-times from 2001 to 2018, accounting for 38.03% of the national total. Among the three provinces in South China, Guangdong had the highest GDP and received the most inbound tourists, and Hong Kong, Macao and Taiwan were the main source of tourists. With the development of the Guangdong–Hong Kong–Macao Greater Bay Area, favorable policies, a planning outline, infrastructure construction, marketing integration and other measures will continue to promote the development of inbound tourism. The southeast region had the second highest value, with the water footprint of inbound tourists accounting for 17.25% of the total. In third place was North China, where the water footprint of inbound tourists accounted for 12.57% of the country’s total.

As regards areas located in the north and south, the water footprint of inbound tourists varied greatly. The contribution rate of the water footprint of inbound tourists in the south was higher than that in the north, with a value of 68.90%, twice that of the north. That is to say, the water consumption of inbound tourists in China was mostly concentrated in the south, while the consumption of the north was relatively small. This was consistent with the spatial distribution pattern of water resources in China and the number of inbound tourists, with more in the south and less in the north.

According to geographical location, economic conditions and the actual economic and technological level and regional differences, the country is divided into three economic zones: the eastern coastal zone; the central zone; and the western zone. According to the calculation, among the three economic zones of east and west, there was no doubt that the eastern zone, which exhibited various convenient conditions, contributed the most to the water footprint of the inbound tourists, accounting for 78.24% of the national total, followed by the central zone, accounting for 11.37%, and the western zone, with a value of only 10.39%. In terms of the water footprint of inbound tourists, the difference between the central and eastern regions was huge, while the difference between the central and western regions was not significant. This also indicated that the development of inbound tourism in the central and western zones had a weak position, while the eastern region was still the core region of inbound tourism development due to the convenient transportation and developed economy as well as other socio-economic factors. The central and western regions are endowed with rich natural and cultural resources, especially ethnic minority cultures, which are of great appeal to international tourists. In the future, we should fully exploit these characteristic tourism resources and encourage the development of inbound tourism.

### 3.3. Spatial-Temporal Evolution

**Figure 3:**
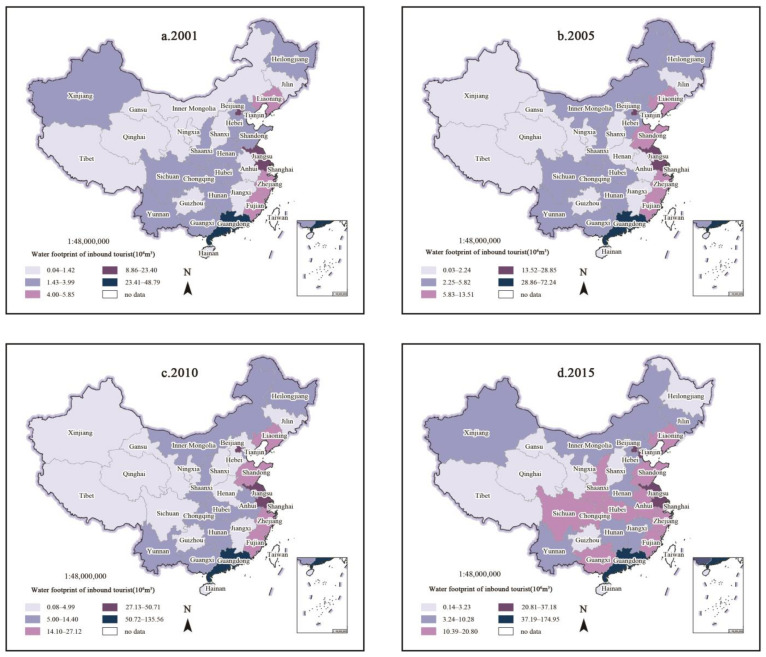

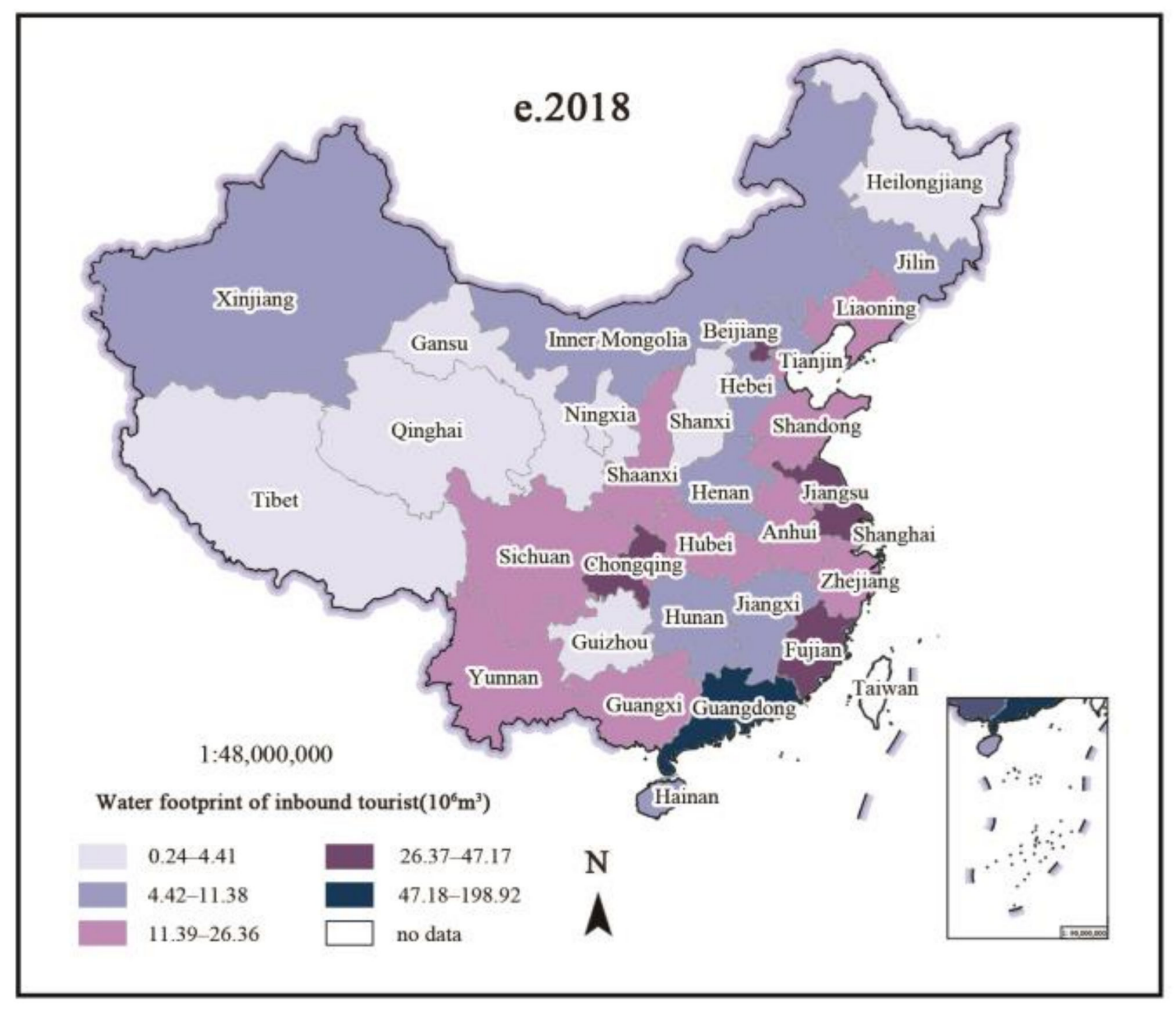
Spatial-temporal evolution pattern of water footprint of inbound tourists from 2001 to 2018.

In order to further analyze the spatial-temporal evolution of water footprints of inbound tourists, Natural Breaks in ArcGIS were used to divide the water footprint from high value to low value into five levels, consisting of high, relatively high, medium, relatively low, and low (Figure 3).

On the whole, the water footprint of inbound tourists decreased from the eastern coast to the inland northwest. The low value area and the relatively low value area showed a decreasing trend, while the medium value area and the relatively high value area displayed a growing trend. Guangdong has always occupied the high value area. The spatial pattern tended to be balanced, and the difference among provinces narrowed over time. This is in agreement with the analysis of the kernel density estimation curve.

To be specific, in 2001, there were more low areas and relatively low value areas, while medium, relatively high, and high value areas were only distributed in six provinces and cities along the eastern coast. In 2001, a small number of provinces and cities along the eastern coast were open to the outside world and had convenient transportation, attracting the majority of inbound tourists. In 2005, there were more low areas and relatively low value areas, concentrated in the central and western regions. Medium, relatively high and high value areas were only distributed in seven provinces and cities along the eastern coast, with little change. Compared with 2001, the number of relatively low value areas increased and developed. In 2010, there were still more low and relatively low value areas, which exhibited little change compared with 2005. In 2015, the most obvious change was the expansion of medium value areas, with some areas in the central and western regions changing from relatively low value areas to medium value areas. Inbound tourism destinations were still dominated by coastal provinces and cities with a high degree of openness, but were gradually shifting to the central and western regions, where cultural resources and ecological resources and environments were superior. This also confirmed the fruitful results of the Strategy for the rise of central China and the Strategy for the large-scale development of western China. These two strategies are major elements in China’s coordinated regional development strategy, and key factors supporting the construction of a moderately prosperous society. This is of vital importance to economic, political, and social development. The continuous influx of capital, information, and technology has improved the infrastructure conditions in the central and western regions, and with rich tourism resources these areas have attracted the attention of international tourists. In 2018, the medium and relatively high value areas were further expanded, while the low and relatively low value areas were still concentrated in the northwest region, representing about half of the provinces. The water footprint of inbound tourists in all provinces was gradually developing from a state of polarization and differentiation to one of equilibrium.

### 3.4. Structural Differences of Virtual Water “Exports”

#### 3.4.1. Differences in Accounts 

Of the three accounts addressed in this study, the contribution rate of the water footprint of food consumption is the largest, accounting for 69.89% of the water footprint of all inbound tourists. Second is the accommodation water footprint (23.06%) and third is the transportation water footprint (7.05%).

(1) Water footprint of inbound tourists’ food consumption.

Food consumption plays an important role in promoting the sustainable development of tourism. Food is one of the most sensitive issues in the tourism industry, and food consumption is one of the core aspects of the tourism and catering industry. It has gradually become a quality symbol and can influence the concept of a vacation. For example, large buffets offering a variety of choices have become a popular part of vacations, guiding tourists’ cognition and perception of destinations and the quality of vacations [59]. In this study, the water footprint of food consumption per capita of the permanent residents in each province was used as a reference to calculate the water footprint of provinces receiving inbound overnight tourists.

The food consumption of inbound overnight tourists in China was 5083.26 × 10^6^ m^3^ from 2001 to 2018, with an annual average of 282.40 × 10^6^ m^3^, and the trend increased year by year, from 110.89 × 10^6^ m^3^ in 2001 to 464.71 × 10^6^ m^3^ in 2018, exhibiting an increase of more than four times. Guangdong had the highest consumption of virtual water for inbound overnight visitors, with 1549.72 × 10^6^ m^3^, followed by Beijing and Jiangsu province with 417.02 × 10^6^ and 414.53 × 10^6^ m^3^, respectively. The food consumption of inbound overnight tourists in the three provinces and cities accounted for 46.85% of the national share. Ningxia, Qinghai, and Gansu displayed the lowest consumption of virtual water for inbound overnight visitors.

**Figure 4:**
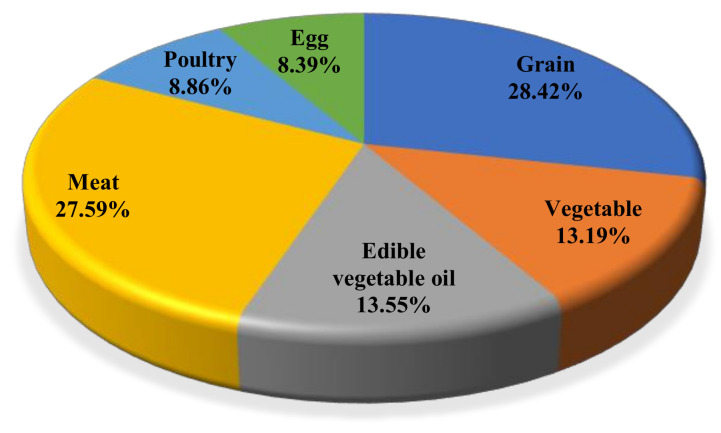
Water footprint of inbound tourists’ food consumption (according to food consumption structure).

Since 2001, the water footprint of food consumption of grain, vegetables, edible vegetable oil, meat, poultry, and eggs has shown an increasing trend, and the increase in poultry, meat, and eggs has been significant. Grain and meat consumption have greatly contributed to the water footprint of inbound tourists’ food consumption, with a contribution rate of 28.42% and 27.59%, respectively, for overall food water footprint. Vegetable oils and vegetables exhibited the next highest values, followed by poultry and eggs (Figure 4).

(2) Accommodation water footprint of inbound tourists.

The accommodation water footprint includes physical water consumption and virtual water consumption. Physical water mainly includes water employed for guest rooms, kitchens, laundry and cleaning, lawns and gardens, swimming pools, etc. Virtual water mainly involves the energy consumption of accommodation facilities. In principle, the measurement of the accommodation water footprint of inbound tourists requires the selection of data on accommodation facilities in each province and the water consumption data of each specific accommodation facility. Detailed data on these two aspects are not recorded. We used the unified standard data proposed by Stefan Gössling, which were calculated based on the average consumption of 0.35 m^3^ physical water and 0.075 m^3^ virtual water by tourists every night [44]. The accommodation water footprint of inbound tourists was the product of the per capita consumption per night, number of days of the stay, and reception volume. From 2001 to 2018, the accommodation water footprint of inbound overnight tourists in China was 1676.91 × 10^6^ m^3^, averaging 93.16 × 10^6^ m^3^ per year. The accommodation water footprint generated by receiving inbound tourists in Guangdong province was the highest, with an annual average of 25.69 × 10^6^ m^3^, followed by Shanghai and Beijing with an annual average of 7.82 × 10^6^ and 7.12 × 10^6^ m^3^, respectively, with the contribution rate of the three provinces and cities reaching 43.61% of the total.

(3) Transportation water footprint of inbound tourists.

Tourism transportation refers to all kinds means provided for tourists’ transfer through space, which can be divided into short distance transportation and long distance transportation, mainly including tourists’ round trips from source areas to destinations and all kinds of transportation facilities and services needed for tourism activities at the destination. These modes or means mainly cover air, rail, road, shipping, and other transportation services. The tourism water footprint of transportation consists mainly of the virtual water implied by transportation energy consumption. Transportation energy is predominantly electricity, gasoline, diesel oil, etc. Inbound travelers have different water footprints, depending on the distance they travel and the means of transportation they choose. However, there are no statistics on the transportation of inbound tourists in China, including the choice of means of transportation and the distance traveled. Therefore, we adopted the unified standard data proposed by Stefan Gössling. The water footprint of tourism transportation was calculated based on the average consumption of 0.13 m^3^ of virtual water by tourists per night [44]. The total water footprints of inbound tourists in each province and the whole country were calculated based on the known time of stay and reception number of inbound tourists. From 2001 to 2018, the transportation water footprint of inbound overnight tourists in China was 512.98 × 10^6^ m^3^, averaging 28.50 × 10^6^ m^3^ per year. Guangdong had the highest transportation water footprint (7.86 × 10^6^ m^3^ per year), followed by Shanghai (2.39 × 10^6^ m^3^ per year) and Beijing (2.18 × 10^6^ m^3^ per year).

#### 3.4.2. Differences in Water Resources Structure

According to source, the water footprint can be divided into green, blue, and gray water footprint [7]. Green and blue water represent the physical water resources in nature. Green water is the “invisible water” absorbed by plants from the soil [12], whilst blue water is visible surface runoff and underground runoff water, mainly used for agricultural irrigation, and industrial and domestic water. Gray water indicates water pollution, which denotes the volume of freshwater that is required to assimilate the load of pollutants based on existing ambient water quality standards, which, to a certain extent, reflects the technical level of agricultural production [13,66], and is also an important indicator employed to explain the quantitative evaluation of water resources, from simple water quantity accounting, to comprehensive water quality and quantity accounting.

Since only the food water footprint of inbound tourists covers the complete green, blue, and gray water spectrum, this section mainly presents the structure of the food water footprint. The results showed that the contribution rate of the green water footprint of inbound tourists’ food consumption was the highest, reaching 72.48%. The second highest value was recorded for the gray water footprint, which accounted for 17.98% of the water footprint of food consumption, and the third highest was obtained for the blue water footprint, accounting for 9.54%.

## 4. Discussion

### 4.1. Influencing Factors

The reasons for the significant difference in the water footprint of inbound tourists at the provincial and regional level are complex and the influencing factors are variable. From the perspective of each province, economic development, tourism infrastructure differences, tourism product differences, tourism resource level, tourism service level, etc., all affect the water footprint. In terms of tourists, the number of inbound tourists, stay time, and the behavioral differences of tourists also have an impact on the water footprint. Among the eight regions, the water footprint contribution rate of inbound tourists in South China was the highest. Of the three provinces in South China, Guangdong has the highest GDP and the highest number of inbound tourists. Its developed economy, convenient transportation, location adjacent to Hong Kong, Macao, and Taiwan, and other advantageous conditions, have all promoted the development of inbound tourism. In recent years, inbound tourism in southwest China has developed rapidly and this has become a typical and key region for its development [67]. The natural and cultural tourism resources in southwest China are abundant, with obvious characteristics that are attractive to inbound tourists. On the whole, among the five provinces and cities in southwest China, Yunnan and Chongqing displayed a relatively high level of inbound tourism development and Sichuan province exhibits a relatively fast growth rate of inbound tourism development, while Tibet and Guizhou have a relatively low level and slow development of inbound tourism. The abundance of tourism resources, the accessibility of a location and transportation, and the level of economic development are the main factors affecting the differences in the development level of inbound tourism among provinces and cities in southwest China [68].

### 4.2. Coordinated Development of Inbound Tourism and Water Resources

The development of inbound tourism not only brings economic benefits, but also generates the “export” of virtual water, affecting the global allocation of water resources. As one of the countries with a shortage of water resources, China must take the problem seriously. This issue requires consideration of how the harmonious unity of inbound tourism and water resources can be realized. Suggestions for each of the three accounts will now be put forward, respectively.

#### 4.2.1. Water and Food

Reducing the food water footprint is essential to reducing the water footprint of overall inbound tourists, and this needs to be considered in a number of ways. From the perspective of the food consumption structure, it is critical to reduce the consumption of grain and meat. For inbound tourists, it is necessary to adhere to the concept of ecological consumption and to balance the diet structure.

In addition, tourists and food service providers should pay attention to reducing food waste. A large amount of food is lost or wasted in production and consumption [69]. In 2010, for example, about 19% of China’s grain was lost or wasted, equivalent to wasting 135 × 10^9^ m^3^ of the water footprint. Consumer waste accounts for the largest part of the total food waste [70]. If people in China were willing to reduce their daily food waste by an average of a spoonful (~5 g), there would be 2.6 million tons more food and an annual saving of 1.79 billion m^3^ in the water footprint [69]. According to this data, the water saving would be about 1.26 m^3^ per person per day. Taking 2018 as an example, the water saving of inbound tourists would be 0.44 billion m^3^. Additionally, the food waste per meal per capita of tourists (96.54g) was higher than that of non-tourists (73.79g) [71]. Therefore, the water saving of inbound tourists would be even higher. From the perspective of tourism catering enterprises, food management should be carried out, including food procurement, preparation and presentation, to reduce the water footprint.

Note that due to the differences in personal consumption habits and food supply in tourist destinations, the food consumption of tourists and residents will vary to some extent. Some studies have found that tourists generally consume more water on vacation than they do at home, and more than locals [72]. Tourists usually use two to three times more water per person than the residents of the destination [73]. In addition, different forms of tourism (e.g., mass tourism vs. luxury tourism) lead to different levels of water consumption. Cheaper forms of tourism tend to exhibit a significantly lower total water consumption [74]. For the specific or micro-tourism destination, it is better to obtain food consumption data through field research.

Furthermore, improving agricultural techniques to reduce the water footprint per unit of food is one of the fundamental measures that can be applied to solve the problem, especially the reduction of green and gray water. Green water is the main source of agricultural production in China, contributing far more than the footprint of blue water and gray water. However, it is often considered unimportant due to the relatively low or negligible opportunity cost [75] and its low negative environmental externalities [76]. It plays an important role in measuring the impact of agricultural production on the water environment [75] and supports plant production in terrestrial ecosystems, including rain-fed farmland [77]. In addition, it affects global agricultural production and food security [76]. Therefore, to reduce the food water footprint, domestic agricultural production should pay attention to green water management and soil and water protection, and minimize the loss of non-productive green water evaporation [78]. Moreover, the proportion of gray water was as high as 17.89%, which indicated that China’s agricultural production involved serious pollution and agricultural technology needs to be improved urgently. Compared with many countries, China’s grain water footprint was very high. From 2001 to 2014, China’s foreign trade data for agricultural products showed that the proportion of China’s exported agricultural products’ gray water footprint reached 21%, while the import value was only 3% [16]. Agricultural trade data on China and One Belt One Road countries showed that the proportion of the gray water footprint of China’s exported agricultural products reached 22.3%, while the import value was only 4.1% [13]. Agricultural production is a big water consumer in China. In 2018, agricultural water consumption was 369.31 × 10^9^ m^3^, accounting for 61.4% of total water consumption and far exceeding that of domestic and industrial water consumption [79]. Additionally, China is facing a serious shortage of water resources. Its agricultural production technology still lags behind that of developed countries to some extent. Therefore, it is very important to introduce advanced technology, especially water-saving irrigation technology. In addition, it is very important to reduce the use of fertilizers and pesticides to prevent water pollution.

#### 4.2.2. Water and Accommodation

How to reduce the water footprint of tourist accommodation requires specific analysis of each water consumption system in the accommodation sector. According to Smith et al., Australian hotels used the most water in their rooms (42%), followed by kitchens (16%), laundry rooms (15%), public toilets (12%), cooling towers (10%), irrigation (3%) and swimming pools (2%) [80]. A study of the Iberotel Sarigeme Park Hotel in Turkey found that kitchens and laundry rooms used the most water (30%), followed by swimming pools (20–25%) and guest rooms (12%) [81]. Generally, the higher the hotel level, the higher the relative water consumption. This is a challenge for accommodation water resource management.

Overall, guest room and laundry water use should not be neglected. Guest room water includes shower and bathtub, toilet flush, and tap water use. The amount of water used may vary depending on the type and standard of hotel (low-budget accommodation may not have a bathtub, while a 5-star hotel may have a Jacuzzi), type of traveler (business, leisure, or vacation), tourist nationality, or cultural or personal preference. This water use can be controlled. For example, tourists can control the frequency and duration of showers and washing, and hotels can choose smart toilets with large and small flushing options [59]. The laundry room needs to clean a large number of items, which will involve a considerable amount of washing, which is an important part of water and energy consumption in hotels. Towels and bedding are the top concerns of customers, as customers’ perception of hotel service expectations has led many hotels to establish a daily towel replacement policy (up to three towels per guest), along with bedding. If tourists are encouraged to use a towel for two days, the amount of washing, energy, and detergent can be reduced by half, and the workload of cleaners can be greatly reduced. Hotels should encourage changing bedding on a per-guest basis rather than on a daily basis. In addition, a descriptive normative approach can be used to encourage visitors to reuse towels or decrease the frequency of changing. This approach is based on the statement “Help us protect the environment”, with additional information about how doing so can contribute to protecting the environment, saving water or detergents [59].

#### 4.2.3. Water and Transportation

Transportation energy utilization is a significant problem for the sustainable development of tourism, and there are few technical solutions available at present. Currently, replacing fossil energy with bioenergy is recommended, but from the point of view of water consumption, this will significantly increase the tourism water footprint, as the amount of water needed for biofuels is two orders of magnitude higher than that for traditional fuels [59]. According to UNESCO, 44 cubic km, or 2% of irrigation water is already used for biofuel production, suggesting that 1L of biofuel production may currently require 2.5 m^3^ of water [82]. If the world’s current commercial aircraft were to use biofuels, an additional 180 cubic km of irrigation water would be required [83]. From the point of view of the water footprint, solar energy, wind energy, geothermal energy, marine energy, and other new energy used to generate electricity is advocated. Biofuels replacing diesel and gasoline will significantly increase water consumption, which has a positive effect on the improvement of the atmospheric environment quality, but is not sustainable for water resources. How to reduce the transportation water footprint of tourists in the future is worth thinking about.

### 4.3. Strengths and Limitations

Virtual water and the water footprint are important factors when studying the environmental impact of water resources. Research mainly focuses on agriculture and industry, and research in the service sector needs to be enriched. The consumption of water resources of inbound tourists in China, from the perspective of trade, is equivalent to China’s “export” of water resources, representing China’s contribution to global water resources. This study presents a new perspective on inbound tourism and water issues. We have put forward suggestions for three specific accounts, conducive to the coordinated development of inbound tourism and water resources. However, limited by data acquisition, this study only measured the three accounts of food, accommodation, and transportation, which consumed large amounts of water, and the overall amount of data was relatively small. In addition, food consumption adopted the data of permanent residents as the reference, water consumption per guest per night of accommodation, and transportation used unified data which neglected the individual differences between tourists. In the future, a larger amount of data will be investigated to strive for the production of more accurate research. Moreover, whilst virtual water trade includes export and import, this study only considered virtual water “exports” generated by Chinese inbound tourists. The “imports” and “exports” of virtual water brought about by outbound and inbound tourists should be comprehensively considered to better serve the development of tourism and optimize the allocation of water resources.

## 5. Conclusions

Virtual water and the water footprint are new perspectives employed to understand water resources. Trade in agricultural and industrial products brings about the flow of virtual water. The flow of people is the most complex, and also brings about the flow of virtual water embedded within it, especially via the cross-border flow of international tourism. Therefore, virtual water flow and trade generated by it will inevitably have an impact on global water resource allocation.

Based on the concept of the water footprint and virtual water, this study analyzed the water footprint of Chinese inbound tourists, namely, the virtual water “exports” from 2001 to 2018. The total virtual water “exports” exhibited a value of 7273.15 × 10^6^ m^3^, with an annual average of 404.06 × 10^6^ m^3^. Overall, the value showed an upward trend and fell twice between 2001 and 2018, in 2003 and 2013, respectively. It varied greatly at the provincial and regional level. From 2001 to 2018, the overall water footprint of inbound tourists in Guangdong province was the highest, reaching 2153.66 × 10^6^ m^3^. Beijing and Jiangsu displayed the next highest values, whilst the lowest were obtained from Ningxia, Qinghai, and Gansu. The contribution rate of the water footprint of inbound tourists in South China was the largest among the eight regions. The value recorded for the south was higher than that in the north, with a value of 68.90%, twice that of the north. Among the three economic zones, the eastern zone contributed the most and accounted for 78.24%. Kernel density estimation and ArcGIS were used to analyze the spatial–temporal evolution. The kernel density curves showed a “single peak”-type feature, indicating that the water footprints of inbound tourists in China were not significantly polarized. However, there was a big difference in some years. ArcGIS spatial analysis showed that the spatial pattern tended to be balanced, and the difference among provinces narrowed over the years. The virtual water “exports” of inbound tourists decreased from the eastern coast to the northwest inland. The low value area and the relatively low value area exhibited a decreasing trend, while the medium value area and the relatively high value area displayed a growing trend. Guangdong always occupied the high value area.

Structural differences were analyzed based on the accounts and water resource structure. The contribution rate of the water footprint of food consumption was the largest, and grain and meat consumption greatly contributed to the water footprint of inbound tourists’ food consumption. In addition, the green water footprint in the food consumption footprint of inbound tourists was the largest. Virtual water “exports” of inbound tourism are bound to have an impact on water resources, so it is necessary to consider how the harmony and unity of ecological and economic benefits can be realized. This study analyzed the influential factors and put forward suggestions for reducing the water footprint on the premise of ensuring tourism quality from the point of view of food production and consumption, tourism accommodation and transportation.

## Figures and Tables

**Table 1:** A summary table of the data used.

Accounts	Data	Resources
food water footprint	food consumption (the consumption of permanent residents as the reference)	China Statistical Yearbook, Statistical Yearbook of Provinces, Statistical Bulletin of National Economy and Social Development of Provinces
	the water footprint of each unit of agricultural products	Report no. 47 and 48 [9,10]
	the number of inbound tourists	China Tourism Statistical Yearbook
	the number of days to stay	China Tourism Statistical Yearbook
accommodation water footprint	water consumption per guest per night	0.425 m^3^ [51]
	the number of inbound tourists	China Tourism Statistical Yearbook
	the number of days to stay	China Tourism Statistical Yearbook
transportation water footprint	water consumption per guest per night	0.13 m^3^ [51]
	the number of inbound tourists	China Tourism Statistical Yearbook
	the number of days to stay	China Tourism Statistical Yearbook

## Data Availability

The data presented in this study are available on request from the corresponding author.
